# A CAMHing Influence: Can a Recruitment Event Inspire Resident Doctors to Train in Child and Adolescent Psychiatry?

**DOI:** 10.1192/bjo.2025.10260

**Published:** 2025-06-20

**Authors:** Hayley Bowes, Lizzy Donaghy, Parvathy Mohandas, Karen Fulton

**Affiliations:** Belfast Health and Social Care Trust, Belfast, United Kingdom

## Abstract

**Aims:** Demand for child and adolescent mental health services (CAMHS) in Northern Ireland (NI) has steadily increased in recent years. Currently, over 50% of families are waiting beyond the recommended nine-week target for assessment. A regional 10-year workforce review outlined the need to expand the CAMHS workforce by 102%, with specific focus on succession planning for psychiatrists. This highlights the need to fill vacant Child and Adolescent Psychiatry (CAP) higher training posts.

By hosting a recruitment event, we aimed to provide information regarding the application process to higher training in CAP and increase exposure to the breadth of career opportunities available. Longer term aims are to sustain recruitment into higher specialty training and bolster the consultant workforce.

**Methods:** The event was delivered as a two hour in-person meeting. Demographic information and anonymous feedback were collected via Microsoft forms. The programme included presentations from current higher trainees, consultants and the Training Programme Director. Topics covered included the following:

Application and interview process.

Structure of training including on-call rota arrangements.

Day-to-day experience of a consultant covering a step three community CAMHS post.

Overview of specialist service provision in CAMHS including eating disorder, autism and CAMHS intellectual disability services.

The event also provided opportunity for networking and an open-ended question and answer session.

**Results:** The event was attended by six local core psychiatry trainees. Four attendees had previously worked in a CAP post, whereas two had no CAP experience or only out of hours exposure to the specialty. All attendees ‘strongly agreed’ that the event had increased their knowledge about the opportunities in CAP in NI. Five attendees ‘agreed’ that the event had strengthened their aspirations to train in CAP with one attendee responding ‘strongly agree’ to this statement.

**Conclusion:** This was the first CAP higher training recruitment event hosted in NI. Although the number of attendees was small, feedback was overwhelmingly positive. Following a year without any regional recruitment into CAP, there have been six applicants for one post advertised for August 2025. Whilst a direct causal relationship cannot be established, we hope that this event reinforced aspirations to pursue this career path. Given the current vacancies across the CAMHS workforce, there is an opportunity to expand this event to include other members of the multi-disciplinary team, to equip the future workforce for the rising demand in services.

